# Supplementary material for: SERS Liquid Biopsy Profiling of Serum for the Diagnosis of Kidney Cancer
Source: Biomedicines. 2022 Jan 22;10(2):233. doi: 10.3390/biomedicines10020233 (PMC8869590; doi:10.3390/biomedicines10020233)
Supplement: Supplementary file 1 [file biomedicines-10-00233-s001.zip › Suplementary Tables S1 S2 S3 S4.pdf]

Supplementary Table S1. Demographic data and tumor related information of the renal cell carcinoma patients enrolled in the study.

Number	Age (years)	Sex	Tumor histology	Tumor grade	TNM stage	Stage
**1**	51	F	Clear cell carcinoma	3	T1bN0M0	1
**2**	60	M	Clear cell carcinoma	1	T1a N0M0	1
**3**	41	M	Clear cell carcinoma	1	T1a N0M0	1
**4**	66	M	Clear cell carcinoma	1	T1a N0M0	1
**5**	59	F	Clear cell carcinoma	1	T1b N0M0	1
**6**	52	M	Clear cell carcinoma	2	T1a N0M0	1
**7**	41	M	Clear cell carcinoma	1	T1a N0M0	1
**8**	63	M	Clear cell carcinoma	1	T1b N0M0	1
**9**	58	F	Clear cell carcinoma	1	T1a N0M0	1
**10**	59	F	Clear cell carcinoma	2	T1a N0M0	1
**11**	50	M	Clear cell carcinoma	3	T2b N0M0	2
**12**	48	M	Clear cell carcinoma	3	T2b N0M0	2
**13**	59	M	Clear cell carcinoma	2	T3aN0M0	3
**14**	66	M	Clear cell carcinoma	2	T3a N0M0	3
**15**	59	F	Clear cell carcinoma	2	T3a N0M0	3
**16**	53	M	Clear cell carcinoma	2	T3a N0M0	3
**17**	49	F	Clear cell carcinoma	1	T3a N0M0	3
**18**	68	F	Clear cell carcinoma	2	T3a N0M0	3
**19**	64	F	Clear cell carcinoma	1	T3a N0M0	3
**20**	64	M	Clear cell carcinoma	2	T3b N0M0	3
**21**	71	M	Clear cell carcinoma	3	T3b N0M0	3
**22**	68	M	Clear cell carcinoma	2	T3a N0M0	3

**23**	46	M	Clear cell carcinoma	2	T3a N0M0	3

Supplementary Table S2. Demographic data of control patients.

Number	Age (years)	Sex
**1**	79	M
**2**	56	M
**3**	59	M
**4**	46	M
**5**	57	M
**6**	71	M
**7**	80	M
**8**	64	F
**9**	70	F
**10**	68	M
**11**	71	M
**12**	36	F
**13**	46	M
**14**	65	M
**15**	41	F
**16**	45	M
**17**	75	M
**18**	45	M
**19**	76	F
**20**	54	M
**21**	37	F
**22**	37	M
**23**	60	M
**24**	58	M
**25**	59	M
**26**	55	F
**27**	58	F

Supplementary Table S3. The performance metrics for the classification of Stage 1 renal cell carcinoma and control group patients based on surface-enhanced Raman scattering (SERS) spectra of serum using three classification algorithms (random forest, kNN and naïve Bayes). PCs 2, 6 and 9 were used as input for the machine learning algorithms. AUC - area under the curve; CA - classification accuracy; F1 - score representing the harmonic mean of precision and recall; Precision - positive predicted value; Recall - sensitivity.

Statistical model	AUC	CA	F1	Precision	Recall
**Random forest**	0.78	0.75	0.76	0.76	0.75
**kNN**	0.73	0.73	0.70	0.70	0.73
**Naïve Bayes**	0.67	0.73	0.70	0.70	0.73

Supplementary Table S4. The performance metrics for the classification of Stage 3 renal cell carcinoma and control group patients based on surface-enhanced Raman scattering (SERS) spectra of serum using three classification algorithms (random forest, kNN and naïve Bayes). PCs 2, 6, 9 and 1? were used as input for the machine learning algorithms. AUC - area under the curve; CA - classification accuracy; F1 - score representing the harmonic mean of precision and recall; Precision - positive predicted value; Recall - sensitivity.

Statistical model	AUC	CA	F1	Precision	Recall
**Random forest**	0.86	0.78	0.77	0.78	0.78
**kNN**	0.72	0.68	0.69	0.70	0.68
**Naïve Bayes**	0.88	0.84	0.84	0.85	0.84

Supplementary Table S5. The performance metrics for the classification of Stage 1 and 3 renal cell carcinoma and control group patients based on surface-enhanced Raman scattering (SERS) spectra of serum using three classification algorithms (random forest, kNN and naïve Bayes). PCs 1 and 9 were used as input for the machine learning algorithms. AUC - area under the curve; CA - classification accuracy; F1- score representing the harmonic mean of precision and recall; Precision - positive predicted value; Recall - sensitivity.

Statistical model	AUC	CA	F1	Precision	Recall
**Random forest**	0.90	0.81	0.81	0.81	0.81
**kNN**	0.66	0.71	0.71	0.72	0.71
**Naïve Bayes**	0.86	0.71	0.71	0.72	0.71
