# Supplementary figures and images for: SERS Liquid Biopsy Profiling of Serum for the Diagnosis of Kidney Cancer
Source: Biomedicines. 2022 Jan 22;10(2):233. doi: 10.3390/biomedicines10020233 (PMC8869590; doi:10.3390/biomedicines10020233)

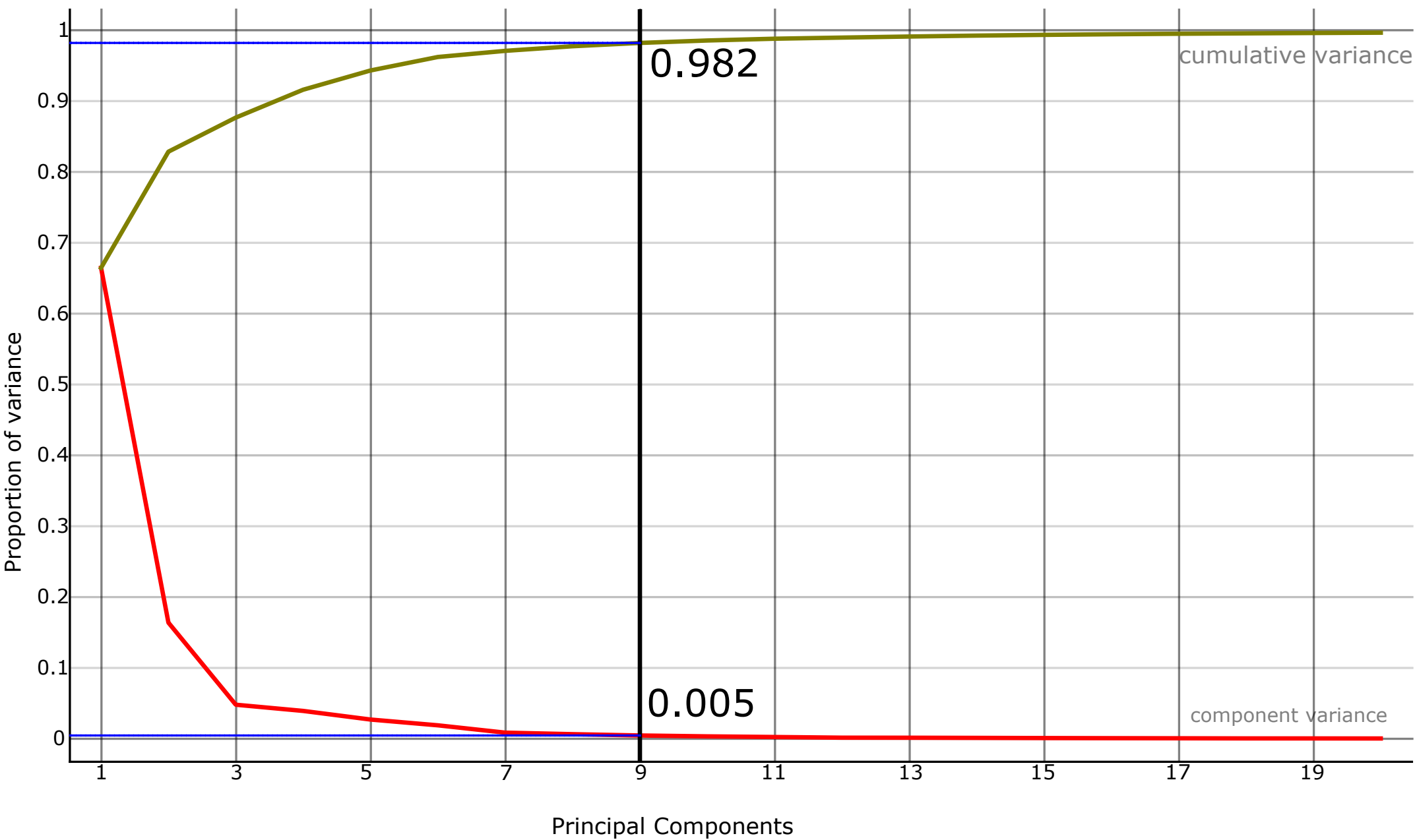

Supplement: Supplementary file 1 [file biomedicines-10-00233-s001.zip › supplementary Figure S1.pdf]
